# Acoustic Signal Recognition of Partial Discharge Optical Fiber Sensors Using Time-Frequency Phase Composition

**DOI:** 10.3390/s26103193

**Published:** 2026-05-18

**Authors:** Xuhui Jin, Pengfei Wang, Pengwei Guo, Xin Liu, Yu Wang

**Affiliations:** 1 Faculty of Natural, Mathematical & Engineering Sciences, King’s College London, London WC2R 2LS, UK; xuhui.jin@kcl.ac.uk; 2 College of Electronic Information Engineering, Taiyuan University of Technology, Taiyuan 030024, China; liuxin01@tyut.edu.cn; 3 College of Electrical and Power Engineering, Taiyuan University of Technology, Taiyuan 030024, China; guopengwei0059@link.tyut.edu.cn; 4 College of Robotics Science and Engineering, Taiyuan University of Technology, Taiyuan 030024, China; wangyu@tyut.edu.cn; 5 Shanxi Key Laboratory of Intelligent Measurement and Control for Coal Mine Electromechanical Equipment, Taiyuan University of Technology, Taiyuan 030024, China

**Keywords:** acoustic emission, deep learning, partial discharge, pattern recognition, time-frequency distribution

## Abstract

A novel method for recognizing acoustic signals of partial discharge optical fiber sensors using the time-frequency phase composition property is proposed in this paper. The method involves obtaining the Wigner–Ville time-frequency distribution for acoustic signals from partial discharge optical fiber sensors through the Cohen bilinear time-frequency transformation, which provides a high time-frequency resolution. The Wigner–Ville distribution could reflect the insulation defect-related properties in detail, owing to the fact that the intensity distribution in the time domain and energy distribution in the frequency domain is seriously influenced by medium dispersion and acoustic propagation. The time-frequency phase composition property is implemented by combining the Wigner–Ville distributions at different phases in the power cycle, which comprehensively represent the characteristics of the acoustic signals from partial discharge optical fiber sensors. A Vision Transformer with an attention block is introduced to identify the acoustic signals of partial discharge sensors. The attention block ensures that the neural network assigns more weight to the energy concentration areas in the extracted acoustic features. To validate the proposed approach, experiments are conducted to identify the acoustic signals of partial discharge optical fiber sensors. The proposed method achieves an impressive accuracy of 99.56% on three group testing sets. This indicates that the proposed approach is a promising method for identifying acoustic signals of partial discharge sensors to detect various insulation defects using acoustic emission feature analysis.

## 1. Introduction

The safe and stable operation of high-voltage power equipment is of utmost importance to ensure the overall reliability of electrical energy production, transmission, and consumption [1]. The insulation condition of power equipment plays a crucial role in ensuring grid security. Unfortunately, insulation defects are inevitable and can lead to partial discharge (PD) occurrences. If these defects are left untreated at an early stage, they can further deteriorate the electrical insulation [2]. Ultimately, the insulation system may fail completely when electric breakdown transpires [3]. Hence, it is essential to focus on PD events and insulation deterioration for the thorough assessment of electrical apparatus [4].

Cross-linked polyethylene (XLPE) power cables play a crucial role in the transmission and distribution of electrical energy due to their exceptional insulation performance, outstanding mechanical properties, and high reliability [5,6]. However, during the processes of manufacturing, transportation, installation, and operation of XLPE power cables, various types of insulation defects may arise, posing different levels of risk to the power system’s safety [7,8]. Additionally, PD activities can provide valuable insights into the condition and characteristics of these insulation defects [9]. Therefore, by employing pattern recognition techniques, different categories of insulation defects can be effectively differentiated based on the distinctive features of PD signals.

The pattern recognition process generally consists of feature extraction and machine learning algorithms. The selection and construction of features play a crucial role in determining the accuracy of the classification, making it the most important aspect of the process [10]. For classifying PD events, the most common approach for feature extraction involves identifying appropriate statistical regulations from the phase-resolved partial discharge (PRPD) variables and pulse current time-domain waveforms during different types of discharge processes [11]. Typical statistical characteristics related to insulation defects, such as wavelet coefficients, discharge phase, discharge variance, etc., were manually selected and combined [12]. These statistical characteristics were then pre-processed to form features that are used to train the machine learning model [13]. However, this kind of feature extraction approach seriously relies on manual selection. It is evident that the addition of other insulation defect types would necessitate the re-selection of statistical characteristics. Consequently, the statistical approach to feature extraction and selection for PD events has inherent limitations to a certain extent.

The PD process is accompanied by various physical phenomena, including partial temperature variation, luminescence, and acoustic emission, apart from the electromagnetic transient phenomena [14]. Acoustic signals are generated as a result of the thermal release during PD activities, indicating a close relationship between the acoustic signal and the PD activity itself. Additionally, the different structures of insulation defects will impact the medium dispersion characteristics and acoustic propagation, ultimately influencing the intensity distribution of waveforms in the time domain and the energy distribution in the frequency domain of the detected acoustic signal [15]. Consequently, the structure feature of insulation defects and PD source characteristics can be reflected through the detected acoustic signals. This has led to widespread interest in and attention to the recognition of PD events based on the acoustic emission feature.

Among various technologies for detecting PD acoustic emission signals, optical fiber sensing has become a research hotspot for the online detection of cable PD due to its unique advantages, such as high-voltage immunity, anti-electromagnetic interference, compact size, and ease of embedding [16]. Researchers have conducted extensive studies on PD signals in cables using various types of optical fiber sensors. PD detection methods based on Fabry–Perot interferometric sensors [17], Fiber Bragg Gratings [18], and interferometric optical fiber sensors [19] offer benefits such as high sensitivity and a wide frequency response. However, their point-sensing characteristics typically limit their application to localized scenarios, such as cable joints and terminals. In contrast, PD detection methods based on distributed optical fiber sensors demonstrate significant advantages in practical long-distance applications due to their continuous and distributed sensing capabilities [20].

The Mel frequency cepstrum coefficient of the detected PD acoustic signals was utilized as a feature, achieving an accuracy of 96.3% using a convolutional neural network [21]. Given the high-frequency nature of the PD acoustic signals, they contain rich details in the high-frequency components. To leverage this, the Hilbert–Huang transformation with fractal feature extraction was employed for PD identification [22]. Moreover, linear time-frequency transformations, such as wavelet and short-time Fourier transformations, were used to extract PD acoustic features [23,24]. Sub-band entropy of the acoustic signal was calculated based on wavelet decomposition, and features were extracted from the sub-band entropy using principal component analysis (PCA). Utilizing the K-nearest neighbor algorithm, an accuracy of approximately 90% was achieved [25]. Additionally, the combination of discrete wavelet transformation with support vector machines led to an accuracy of 98% [26]. Furthermore, the application of multi-scale wavelet analysis with a backpropagation neural network resulted in an accuracy of 96% [27]. Overall, various feature extraction methods and machine learning techniques have been employed to achieve high accuracy in PD event identification based on acoustic emission features.

Indeed, the time-frequency analysis has proven to be the most effective approach for feature extraction, enabling pattern recognition with high accuracy and avoiding manual feature selection to some extent. However, due to the tiny structural size of insulation defects, the time-frequency features show minimal variation. This poses a challenge for linear time-frequency transformations like wavelet and the short-time Fourier transform, as their resolution might not be sufficient for precise analysis of PD acoustic signals [28]. Moreover, linear time-frequency transformations could not reflect the energy distribution of the acoustic signals, and excessive post-processing of time-frequency results might neglect valuable hidden information. Since the energy distribution of PD acoustic signals in the time-frequency domain tends to be concentrated, the machine learning algorithm should pay more attention to these areas. Effectively capturing and leveraging the energy distribution patterns in the time-frequency domain would be a valuable direction to improve the accuracy of PD pattern recognition.

In this paper, a novel approach for high-accuracy identification of PD events by combining bilinear time-frequency feature extraction with an attention mechanism deep neural network is proposed. The Wigner–Ville distribution of the detected PD acoustic signal is obtained using the Cohen bilinear time-frequency transformation. The energy distribution in time-frequency domains with a high resolution could be acquired. The time-frequency phase composition feature involves combining the Wigner–Ville distributions of PD acoustic signals at different discharge phase. Vision Transformer (ViT) with multi-head attention is employed as the neural network for the pattern recognition [29]. To validate our approach, we establish a high-voltage testing platform and an optical fiber sensing system to collect three categories of PD acoustic signals in XLPE power cables. Finally, the efficiency of the proposed method is verified and analyzed with the collected data.

## 2. Principle

### 2.1. Typical Insulation Defects

**Figure 1:**
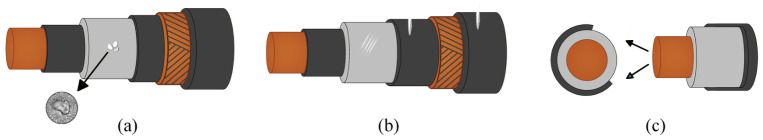
Three typical insulation defects in experiment: (**a**) void defect; (**b**) insulation scratching; (**c**) partial absence of a semi-conductive layer.

Three typical insulation defects are created on an 8.7/15 kV XLPE power cable to simulate various PD activities. These defects include void defect, insulation scratching, and partial absence of the semi-conductor layer. The insulation defects are shown in Figure 1.

The void discharge is typically generated by electrical treeing or tiny insulation defects. In our experiments, we simulate the void discharge by using a steel needle to puncture a hole in the XLPE power cable. The XLPE insulation has an approximate thickness of 4.5 mm, and we carefully insert a steel needle with a diameter of 1 mm to create a 2.5 mm deep air gap in the power cable, as depicted in Figure 1a.

The insulation scratched defect is generally caused by mechanical damage or the installation process. To replicate this defect in our experiments, we used a saw blade to create a scratch with a depth of 1.5 mm on the XLPE power cable, as depicted in Figure 1b.

Surface discharge is commonly observed in the joints or terminals of power cables and is often caused by inadequate treatment of the XLPE insulation and semi-conductive layer surfaces. To simulate surface discharge, we peeled off a part of the semi-conductive layer, as shown in Figure 1c.

As seen in Figure 1, the potential PD acoustic sources and propagation structures differ for the three typical insulation defects. Consequently, the time-frequency distribution of the detected acoustic signals is expected to exhibit distinct characteristics for each type of defect.

### 2.2. High Voltage Apparatus and PD Detection Set-Up

**Figure 2:**
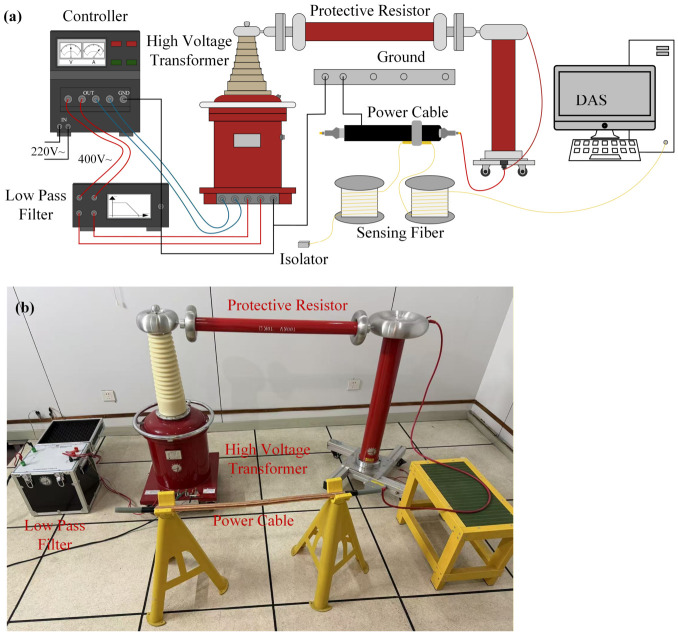
High voltage apparatus for power cable: (**a**) the sketch map of the experiment structure; (**b**) practical apparatus.

The high voltage apparatus used for the power cable PD experiments is illustrated in Figure 2. The high voltage control console is powered by 220 V alternating current (AC). The output range of 0–400 V is adjusted using the control console and then passed through a low-pass filter to eliminate high-order harmonics present in the power grid. The filtered AC power is then stepped up to a voltage range of 0–100 kV by a PD-free transformer, which serves as the high voltage AC source for the power cable experiments. To ensure safety during the experiments, the power cable sample is connected to the PD-free transformer through a protective resistor. This resistor acts as a safeguard against any potential short circuits between the high voltage AC and the ground.

To apply the high voltage to the cable sample, approximately 3 cm of the insulation layer was stripped from one end of the cable to expose the internal copper conductor, which served as the connection node for the high-voltage electrode. Additionally, to prevent surface discharges between the high-voltage conductor and the grounded outer semi-conductive layer, the outer semi-conductive layer was stripped back by more than 10 cm to provide sufficient insulation distance. During the experiment, a step-up voltage method was employed to initiate PD activities. The voltage was increased in increments of 0.5 kV, with each level maintained for 3 min to allow the discharge to stabilize. This process continued until a stable and continuous partial discharge phenomenon was observed and recorded.

**Figure 3:**
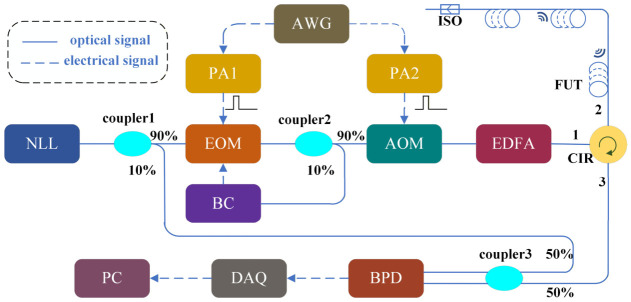
Distributed acoustic sensing system for power cable PD detection.

In this paper, the optical fiber sensing technology is utilized to acquire the PD acoustic signals. Optical fiber serves as both the sensing and transmission unit, offering advantages such as anti-electromagnetic interference, flexibility, and passivity [30]. To capture PD ultrasonic signals directly, the sensing fiber is installed on the insulation defect, as shown in Figure 2. The acoustic signal is modulated on the phase of the light wave and subsequently acquired by a demodulator. In our experiments, we employ a distributed acoustic sensing (DAS) system as the demodulator, which provides benefits such as a long sensing range and independent multi-point sensing capabilities [31]. The DAS setup is illustrated in Figure 3 for PD detection.

A continuous light with a central wavelength of 1550.12 nm is generated by a narrow linewidth laser (NLL). This light is then divided into two branches using a polarization-maintaining optical coupler (OC1). Roughly 10% of the light is directed at the local oscillator light, while the remaining 90% serves as the probe light. Due to the ultrasonic nature of the PD signals, a higher sampling rate is required for the PD acoustic signal. Therefore, frequency division multiplexing technology is applied. The probe light is modulated into a series of frequency carrier lights using an electro-optical modulator (EOM) to enhance the frequency response bandwidth.

A multi-frequency excitation signal is first produced by an arbitrary waveform generator (AWG), and this signal is amplified via a power amplifier (PA1) to drive the electro-optic modulator (EOM). The multi-frequency probe beam is subsequently divided into two branches by a polarization-maintaining optical coupler (OC2). A small portion (1%) of the optical power is returned to the bias controller (BC) to lock the EOM at its optimum working point, while the other 99% is transmitted to an acousto-optic modulator (AOM) that provides a 200 MHz frequency shift. The AOM outputs probe pulses with a repetition rate of 20 kHz and a pulse duration of 160 ns. Before the pulses are launched into the sensing fiber through port 2 of the optical circulator (CIR), an erbium-doped fiber amplifier (EDFA) is employed to enhance the pulse power level.

The total length of the sensing fiber is approximately 4.5 km, comprising a 4 km and a 500 m section dedicated to the experimental setup. This total length ensures that the optical pulse round-trip time remains within the limits required by the 20 kHz pulse repetition rate. To improve the sensitivity to weak PD acoustic signals, a 5 m length of the sensing fiber is wound around the power cable near the insulation defect, creating a concentrated sensing zone. Although the SNR in DAS systems can be affected by fiber length due to exponential pulse energy attenuation—an effect that typically necessitates consideration for distances over 20 km—such attenuation is negligible within the 5 km range used here. Consequently, a stable and high SNR is maintained across the entire sensing fiber in this study.

The PD acoustic signal modulates the phase of the Rayleigh backscattered (RBS) light linearly. This modulated RBS light returns through port 3 of CIR and interferes with the local light in the optical coupler (OC3). The resulting beat signal is then converted into an electrical signal using a balanced photodetector (BPD). Finally, the beat data is collected by a data acquisition card with a sampling rate of 1 GSa/s and a resolution of 14 bits.

The obtained beat signal *I*(*t*) in one detection period could be represented as:

(1) $$I(t)\propto \frac{1}{\sqrt{N}}\sum_{i=1}^{N}A_{i}\cos\left[2\pi f_{i}\left(t-\left(i-1\right)\tau_{s}\right)+\Phi_{i}\right]$$

where *N* is the multiplexing channel number, which could intensify the response bandwidth. *A_i_* is the intensity of the beat signal, *f_i_* is the carrier light frequency which is decided by the EOM and the center frequency of AOM. *τ_s_* is the time interval of the adjacent probe pulse, Φ*_i_* is the phase variation along with the sensing fiber.

The PD acoustic signal is contained in the phase Φ*_i_*. To obtain this phase term, a bandpass filter is used to separate the multiplexing frequency. For one of the channels, the Hilbert transform is initially applied. Subsequently, quadrature demodulation is conducted, yielding the phase component Φ*_i_*. Due to the phase accumulation along the fiber, the *i*th channel’s acoustic signal can be extracted:

(2) $$s_{i}\left(t,z_{0}\right)=\Phi_{i,z_{0}+\Delta d/2}-\Phi_{i,z_{0}-\Delta d/2}$$

where *z*_0_ is the position of the insulation defect, Δ*d* is the length of the fiber optic probe.

In order to obtain a complete acoustic signal in one detection period, the results from each channel need to be combined. The acoustic signal can be represented as *s*(*t*) = [*s*_1_(*t*), s_2_(*t*), …, *s_N_*(*t*)], where *s_i_*(*t*), is the signals acquired from each individual channel. By combining the results from all channels in this manner, the PD acoustic signals generated by the insulation defects can be accurately collected.

### 2.3. Partial Discharge Wigner–Ville Distribution Using Cohen Bilinear Time-Frequency Transformation

The detected PD acoustic signals s(t) typically exhibit non-stationary characteristics. The conventional linear time-frequency transformations encounter the issue of mutual constraints between time and frequency resolution. To address this limitation, the bilinear time-frequency transformation is employed to describe the PD acoustic signals. Compared with linear time-frequency methods such as the short-time Fourier transform and wavelet transform, the Cohen-class bilinear time-frequency transformation provides higher time-frequency resolution for non-stationary PD acoustic signals. Since PD acoustic emissions usually exhibit short-duration ultrasonic characteristics with concentrated energy distributions, high-resolution time-frequency analysis is beneficial for distinguishing subtle differences among various insulation defects. The ZAM kernel was selected in this work because it effectively suppresses cross-term interference while preserving the localized energy concentration characteristics of the Wigner–Ville distribution.

The instantaneous autocorrelation of the detected PD acoustic signals can be defined as follows:

(3) $$R_{s}\left(t,\tau \right)=s\left(t+\frac{\tau }{2}\right)s^{*}\left(t-\frac{\tau }{2}\right)$$

where *τ* is the introduced time delay. s*(**·**) is the conjugate function of *s*(**·**).

By applying Fourier transformation to the autocorrelation function *R_s_*(*t*, *τ*), the Wigner–Ville distribution *WVD_S_*(*t*, *ω*) can be derived. Since the time-bandwidth product of the Wigner–Ville distribution attains the minimum limit defined by the Heisenberg uncertainty principle, it delivers superior time-frequency resolution compared with other conventional joint time-frequency representations [32].

Nevertheless, cross-term interference arising from distinct signal components in the Wigner–Ville distribution will severely distort the time-frequency analysis outcomes. For this reason, the Cohen class bilinear time-frequency distribution is adopted in the present study. Therefore, the Cohen bilinear time-frequency distribution is applied in this paper:

(4) $$C_{s}\left(t,\omega \right)=∭R_{s}\left(u,\tau \right)\gamma \left(\tau ,v\right)e^{-j\left(tv+\omega \tau -uv\right)}dud\tau dv$$

where the *γ*(*τ*, *v*) is the kernel function.

By employing a suitable kernel function, it becomes possible to perform time-frequency smoothing filtering on the Wigner–Ville distribution, effectively suppressing the cross-terms. In this paper, the Zhao–Atlas–Marks (ZAM) kernel function is chosen for this purpose:

(5) $$\gamma \left(\tau ,v\right)=\left|\tau \right|\frac{\sin\left(\alpha \tau v\right)}{\alpha \tau v}w\left(\tau \right)$$

where *α* is the parameters, *w*(*τ*) is the window function. The Hamming window function is used in this paper:

(6) $$w\left(\tau \right)=a_{0}-(1-a_{0})\cos\left(\frac{2\pi \tau }{M-1}\right),\;\;0\leq \tau \leq M-1$$

where *a*_0_ ≈ 0.54. *M* is the window length.

**Figure 4:**
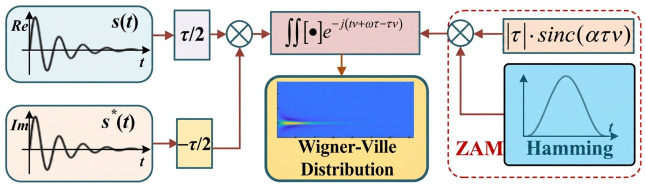
The process of Wigner–Ville distribution using Cohen bilinear time-frequency transformation for PD acoustic signals.

Figure 4 illustrates the steps involved in the Cohen bilinear time-frequency transformation. Initially, the acquired PD acoustic signal undergoes a conjugate transformation. Following this, time delays of ±*τ*/2 are applied, resulting in the instantaneous autocorrelation function. Next, the ZAM kernel function is constructed using the Hamming window function, and the window length is set to 32. Finally, the autocorrelation function is multiplied by the kernel function and then subjected to Fourier transformation, yielding the Wigner–Ville distribution. This transformation process effectively suppresses the cross-terms and provides a time-frequency representation with enhanced accuracy for the PD acoustic signals.

Moreover, the residual cross-term interference after kernel smoothing generally exhibits dispersed and irregular distributions in the time-frequency domain. In contrast, the intrinsic PD acoustic features usually present concentrated energy patterns. Therefore, the attention mechanism in the ViT architecture can further suppress the influence of residual interference components by assigning higher weights to the energy concentration regions during feature learning.

### 2.4. Visual Transformer Neural Network Framework

**Figure 5:**
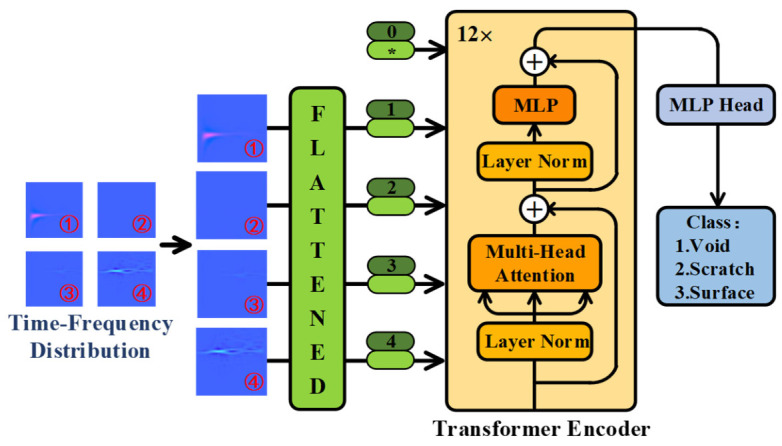
Visual transformer neural network framework.

PD acoustic signals typically have short durations and concentrated frequency distributions. The Wigner–Ville distribution of these signals often exhibits local energy concentration, resulting in many invalid values across most areas of the distribution. Only a few patches in the distribution are worth paying attention to. To address this, the ViT neural network, which incorporates the attention mechanism, is employed for PD acoustic event recognition. The attention block in the ViT neural network allows for effective learning of the energy concentration component of the PD Wigner–Ville distribution. This enhances the accuracy of the classification method significantly. The framework of the ViT neural network is depicted in Figure 5. Here, the * in the 0-th box of Figure 5 denotes randomly generated global parameters, while the blank spaces in boxes 1 to 4 represent the input data.

The framework of the ViT neural network mainly consists of three parts: the embedding layer (flattened patches), transformer encoder, and multi-layer perceptron (MLP) head. The extracted feature is first divided into a set of patches. Each patch is flattened into a one-dimensional vector through linear projection by the embedding layer, resulting in what is referred to as a “token.” Since the position of the patches cannot be neglected, positional encoding is concatenated with each token. After that, the tokens are input into the transformer encoder.

**Figure 6:**
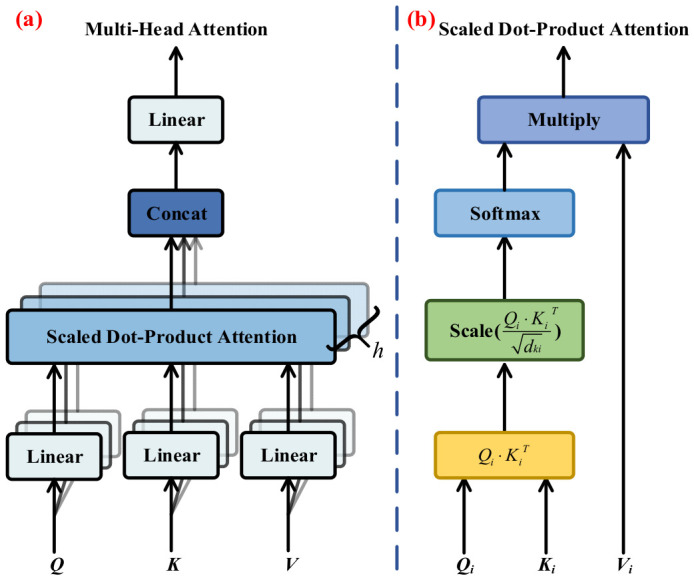
Multi-head attention and scaled dot-product attention structure: (**a**) detailed structure of multi-head attention; (**b**) structure of scaled dot-product attention.

The transformer encoder is primarily composed of a multi-head attention module and a multilayer perceptron (MLP) module. The two modules are linked via residual connections, and layer normalization is introduced to facilitate stable gradient propagation within the network. Multi-head attention is utilized to assign higher weights to informative tokens, representing the core component of the Vision Transformer (ViT) architecture. The detailed structure of the multi-head attention can be found in Figure 6.

Multi-head attention is introduced to strengthen the feature expression capability of the model, as it can capture contextual information from multiple distinct feature subspaces across different spatial locations in parallel [33]. As illustrated in Figure 6a, the final output of multi-head attention is generated by concatenating the outputs of multiple independent self-attention branches followed by a linear projection operation. In this framework, *Q*, *K*, and *V* represent the query, key, and value, respectively, after linear transformations *XW^Q^*, *XW^K^*, and *XW^V^*. Here, *X* refers to the input data, and *W^Q^*, *W^K^*, and *W^V^* are the weight matrices. Therefore, the output result can be represented as follows:

(7) $$\begin{array}{c} \mathrm{MultiHead}(Q,K,V)=\mathrm{Concat}\left(head_{1},\ldots ,head_{h}\right)W^{O}\\ \mathrm{where}\;\;\;\;head_{i}=\mathrm{Attention}\left(Q_{i},K_{i},V_{i}\right) \end{array}$$

where *W^O^* is the linear transformation matrix. Attention(·) is the self-attention module shown in Figure 6b. The self-attention is defined as:

(8) $$\mathrm{Attention}(Q_{i},K_{i},V_{i})=\mathrm{softmax}(\frac{Q_{i}{K_{i}}^{T}}{\sqrt{d_{ki}}})V_{i}$$

where *d_ki_* is the dimensional of the matrix *K* in the *i*th self-attention module.

**Figure 7:**
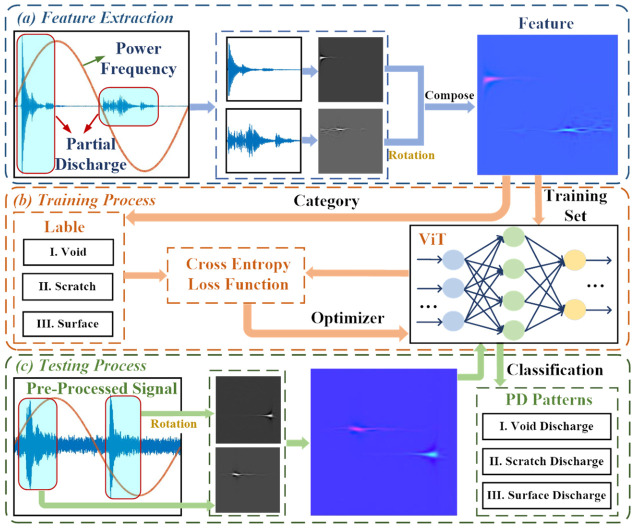
The flowchart for power cable PD recognition: (**a**) feature extraction process; (**b**) training process; (**c**) testing process.

In the training process, the attention block redistributes the weight to the energy concentration areas. The output of the multi-head attention is then passed through the MLP block for training and abstraction, followed by layer normalization, as shown in Figure 5. As the dimensional of the transformer encoder remains unchanged, the 12 blocks of the encoder are cascaded directly to achieve the original data encoding. Finally, the output of the transformer encoder is processed by the MLP head for the classification of the PD acoustic signals. The complete process of power cable PD pattern recognition is depicted in Figure 7. The identification process of PD events consists of three sections: feature extraction, training process, and testing process.

The feature extraction section is illustrated in Figure 7a. The detected PD acoustic signal is first filtered by the wavelet. Then, the Wigner–Ville distribution is obtained using the Cohen bilinear time-frequency transformation. To fully represent the discharge pattern of the entire power frequency at different phase, the Wigner–Ville distribution of the negative half-cycle discharge is rotated 180 degrees to compose the time-frequency phase composition feature for the neural networks.

The training process is depicted in Figure 7b. The constructed training data, along with their corresponding labels, forms the training set. The ViT neural network is utilized for PD recognition in this process. The cross-entropy loss function is selected for the training process:

(9) $$Loss=-\frac{1}{N_{c}}\sum_{i}\sum_{c=1}^{M_{c}}y_{ic}\log\left(p_{ic}\right)$$

where *N_c_* is the sample number. *M_c_* is the number of categories. *p_ic_* is the prediction probability of sample *i* belonging to category *c*. *y_ic_* is the sign function, if sample *i* is category *c*, *y_ic_* = 1, otherwise *y_ic_* = 0.

During the training process, the weights of the neural network are adjusted to enable the classification function. The testing process is presented in Figure 7c. In this process, the feature extraction is performed on the PD acoustic signal, and then the extracted feature is input to the trained neural network. As a result, the neural network accurately determines the category of the PD events.

## 3. Experiment, Results and Discussion

### 3.1. Feature Extraction Results

Three types of insulation defects were made manually for XLPE power cables as represented in Section 2.1. The DAS system is used to obtain PD acoustic signals at insulation defects.

**Figure 8:**
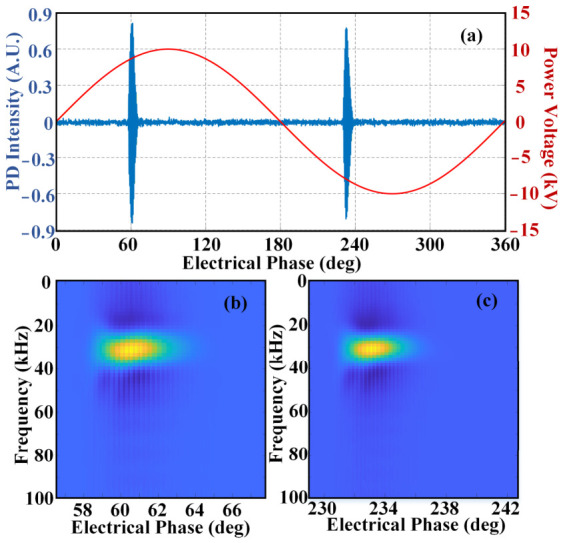
PD activity with void defect: (**a**) time-domain waveform; (**b**) Wigner–Ville distribution of positive half-cycle; (**c**) Wigner–Ville distribution of negative half-cycle.

In the case of a void defect in the XLPE power cable, Figure 8 displays the detected PD acoustic waveform and the corresponding Wigner–Ville distribution. Figure 8a shows the PD acoustic waveform in the time-domain within one power cycle. PD activities occur at positions around 60 deg and 233 deg, respectively. The PD acoustic signal of the void defect exhibits a regular single discharge. The Wigner–Ville distribution result of the positive half-cycle is presented in Figure 8b. The transformation results in a gray matrix used for the neural network. In this part, the color in Figure 8b represents the distribution of acoustic energy. It is evident that the PD acoustic energy is mainly concentrated around 30 kHz with a short duration. Similarly, Figure 8c shows the Wigner–Ville distribution result of the negative half-cycle, also exhibiting energy concentration in the acoustic signal. Therefore, the time domain waveform and the Wigner–Ville distribution for PD activities with a void defect in the XLPE power cable present extremely similar characteristics within positive and negative cycles.

**Figure 9:**
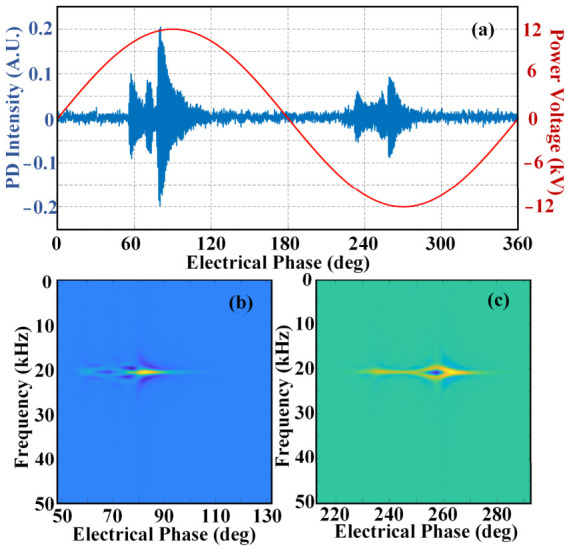
PD activity with scratched defect: (**a**) time-domain waveform; (**b**) Wigner–Ville distribution of positive half-cycle; (**c**) Wigner–Ville distribution of negative half-cycle.

Figure 9 presents the measured PD acoustic signal characteristics of XLPE cables with insulation scratch defects. The time-domain waveform of the PD acoustic signal is plotted in Figure 9a. The signal amplitude of this discharge type is noticeably lower than that of void discharge. Sustained PD events take place in both positive and negative half-cycles of the power frequency, with the signal phase range spanning 60~120 deg in the positive half-cycle and 230~280 deg in the negative half-cycle. The Wigner–Ville distribution of the positive half-cycle signal is given in Figure 9b, where the acoustic energy is primarily concentrated at approximately 20 kHz. Different from void discharge, the acoustic energy shows obvious dispersion characteristics, and multiple low-energy regions (marked in blue) appear with the change in electrical phase. Correspondingly, the Wigner–Ville distribution of the negative half-cycle signal in Figure 9c also presents energy dispersion. This feature is closely associated with the geometric structure of the insulation defect. Since the scratch defect has a rougher structure than the void defect, the acquired acoustic signals are more likely to produce energy dispersion, as reflected in Figure 9b,c.

**Figure 10:**
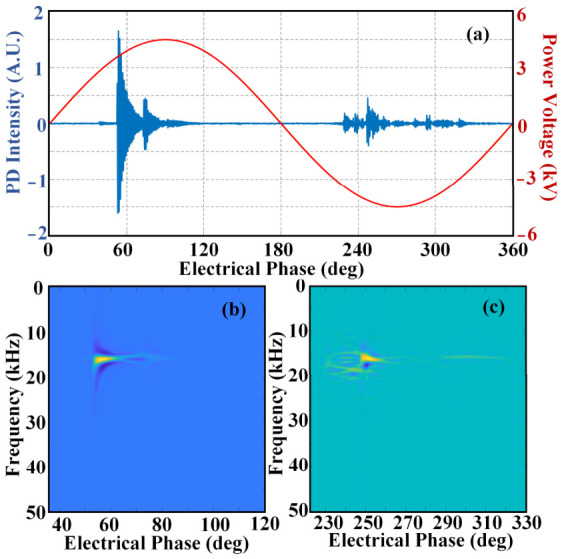
PD activity with surface discharge: (**a**) time-domain waveform; (**b**) Wigner–Ville distribution of positive half-cycle; (**c**) Wigner–Ville distribution of negative half-cycle.

The PD acoustic signal features of XLPE cable surface discharge are demonstrated in Figure 10. Figure 10a shows the time-domain waveform of the corresponding signal. The PD acoustic signals in the positive half-cycle feature higher amplitude and lower repetition frequency, whereas those in the negative half-cycle show lower amplitude and higher repetition frequency. The signal phase interval is about 50~70 deg in the positive half-cycle and 230~320 deg in the negative half-cycle. The Wigner–Ville distributions of the positive and negative half-cycle signals are exhibited separately. The degree of energy dispersion is significantly higher than that of the other two defect types, which is mainly attributed to the large number of discharge sources generated in surface discharge. Overall, the PD acoustic signals of XLPE cable surface discharge present unique traits in both time-domain waveform and Wigner–Ville distribution, with clear discrepancies in signal intensity and repetition between the positive and negative half-cycles.

**Figure 11:**
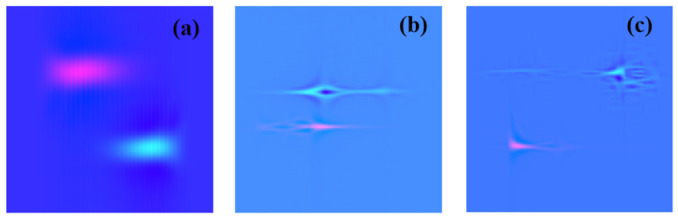
Features of the three PD events: (**a**) void discharge; (**b**) scratch discharge; (**c**) surface discharge.

Indeed, the Wigner–Ville distribution of the PD acoustic signal exhibits blank areas due to the energy concentration of the signal. Additionally, understanding the regulation of PD activities throughout the entire power cycle is crucial. Therefore, the proposed feature extraction method combines the Wigner–Ville distributions from both the positive and negative half-cycles, providing a more comprehensive input to the neural network. The extracted time-frequency composition features for the three discharge events are presented in Figure 11.

Notably, there are evident differences in the features of the three PD events. For the PD event with a void in the cable, the feature appears relatively regular, and the energy is concentrated. On the other hand, for the PD event with scratched defects, energy dispersion occurs, and for the PD event with surface discharge, the energy dispersion is even more pronounced. These distinctions in features allow the proposed method to effectively distinguish between the three PD events. Based on these observations, it is reasonable to utilize this feature extraction method as the input for the neural network in the PD identification process. The ability of the proposed method to capture and differentiate the unique characteristics of each PD event suggests its potential for accurate and reliable PD pattern recognition. The extracted time-frequency features are normalized and resized to 256 × 256 using image processing operations (imresize) to ensure compatibility with the ViT input format. It should be noted that the resulting feature maps represent processed time-frequency matrices rather than conventional plots with explicit physical axis units.

### 3.2. Classification Results

During the neural network training and testing, we utilized a computer equipped with a Core i9-9900K CPU and an NVIDIA GeForce RTX 2080Ti GPU. The training process took approximately 2 h to complete 20 epochs, with a batch size of 16, which consisted of around 18,000 data pairs. For the three types of insulation defects examined in this paper, we employed three power cable samples to generate the training data. Each sample representing one defect contained 2000 pairs of data, resulting in a total of 18,000 pairs of data for the training set, as illustrated in Table 1.

In this paper, the proposed method involves extracting time-frequency features from the PD signals to create the training set. Subsequently, the training set is fed into the ViT network for training, resulting in the formation of the trained network. To validate the neural network’s performance, three groups of testing sets and one group of trained sets are constructed. Each data group comprises 600 pairs of data, with 200 pairs of data for each defect.

To enhance the robustness and generalization capability of the proposed Vision Transformer (ViT) model, an online data augmentation strategy was applied during training. Specifically, Gaussian noise was randomly added to each mini-batch of input time-frequency features to simulate stochastic disturbances in practical operating environments. In addition, random interference perturbations and symmetric transformations were employed to increase the diversity of the training samples.

Since PD acoustic emissions are mainly ultrasonic signals, high-pass filtering was applied during signal preprocessing to suppress low-frequency environmental noise and structural vibration interference. This preprocessing step effectively improves the signal quality before time-frequency feature extraction.

**Figure 12:**
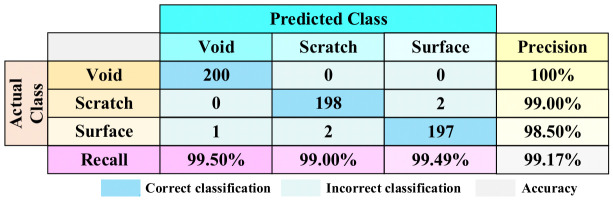
Confusion matrix for the first group testing set.

For the first group testing set, the confusion matrix is shown in Figure 12. The confusion matrix provides an overview of how well the neural network performs in classifying the different PD events. It shows the correct and incorrect classifications for each defect type, allowing for an assessment of the network’s accuracy and performance.

In the confusion matrix for the first group testing set, the first column represents the actual categories of the PD events, while the first row indicates the predicted categories by the neural network. The results show that out of the 200 testing data for the void discharge, all predictions correctly belong to the actual category. For the scratch discharge, 198 data are correctly classified, but two data are incorrectly classified as surface discharge. Similarly, 197 data for the surface discharge are correctly classified. The precisions for the three types of insulation defects are 100% for void discharge, 99.00% for scratch discharge, and 98.50% for surface discharge, respectively, in the first group testing set. The recall rates are 99.50% for void discharge, 99.00% for scratch discharge, and 99.49% for surface discharge. Overall, the void insulation defect shows the highest accuracy and is not easily confused with the other defects. The accuracy of the first group testing set reaches 99.17%.

Table 2 presents the overall metrics results of the proposed method on the training set (S0) and three group testing sets (S1, S2, and S3). The accuracy of the neural network on the training set is an impressive 99.83%, with only one sample being incorrectly classified, indicating excellent performance on the training data.

For the three group testing sets, the accuracies are 99.17%, 99.83%, and 99.67%, respectively, indicating that the neural network is highly effective in completing classification tasks. The average metrics of the three group testing sets are also calculated. The average precisions are 100%, 99.50%, and 99.17% for void discharge, scratched defect, and surface discharge, respectively. The recall rates are 99.83%, 99.33%, and 99.66% for the same categories, respectively. Overall, the accuracy of the neural network achieves an extremely high value of 99.56%, further demonstrating the effectiveness of the proposed method in PD pattern recognition and classification.

Therefore, owing to the PD acoustic signal being related to the insulation defects, the feature extraction method proposed in this paper can clearly distinguish between different insulation defects. By cooperating with the neural network based on the attention mechanism, the accurate classification and identification of PD events for various insulation defects can be achieved.

The proposed Vision Transformer (ViT) model consists of 12 transformer encoder layers, resulting in approximately 10^7 trainable parameters. Although the computational complexity is higher than traditional machine learning methods such as SVM, the proposed model provides significantly improved feature extraction capability for complex time-frequency representations, achieving a favorable trade-off between accuracy and computational cost.

## 4. Conclusions

The paper presents a novel method for partial discharge recognition in XLPE power cables using the time-frequency phase composition of acoustic emission features. The method is based on the understanding that insulation defects in power cables affect medium dispersion and acoustic propagation, resulting in distinct intensity and energy distributions in the time and frequency domains of the detected acoustic signal. To capture these characteristics effectively, the Cohen bilinear time-frequency transformation is employed, which provides a high time-frequency resolution. The application of the ZAM kernel function further realizes the time-frequency smoothing filtering and suppresses cross-terms in the Wigner–Ville distribution. By combining the Wigner–Ville distribution in positive and negative half-cycles at different discharge phase, the PD acoustic signal’s time-frequency phase composition feature is obtained and used as input to the ViT neural network. The attention block in the ViT neural network assigns higher weights to energy concentration areas in the extracted feature, leading to accurate PD event recognition during the training process. The validation experiments were conducted using 18,000 pairs of data representing three types of insulation defects: void defects, scratch defects, and partial absence of a semiconductor layer. The neural network was trained, and three groups of testing data, each containing 600 data, were used to validate the proposed method. The results demonstrate high precision and recall rates for each defect type. The overall accuracy achieved on the three groups of testing set is 99.56%. In conclusion, the proposed PD recognition method based on acoustic emission feature analysis with the Wigner–Ville distribution is a promising approach for identifying various insulation defects in XLPE power cables accurately.

## Figures and Tables

**Table 1:** Composition structure of training set.

Discharge Type	Sample Cable	Number
Void	Sample #1	2000
	Sample #2	2000
	Sample #3	2000
Scratch	Sample #1	2000
	Sample #2	2000
	Sample #3	2000
Surface	Sample #1	2000
	Sample #2	2000
	Sample #3	2000

**Table 2:** The classification results of the neural network.

		Void	Scratch	Surface	Accuracy
S0	Precision	100%	99.50%	100%	99.83%
	Recall	100%	100%	99.50%	
S1	Precision	100%	99.00%	98.50%	99.17%
	Recall	99.50%	99.00%	99.49%	
S2	Precision	100%	100%	99.50%	99.83%
	Recall	100%	99.50%	100%	
S3	Precision	100%	99.50%	99.50%	99.67%
	Recall	100%	99.50%	99.50%	
AVG	Precision	100%	99.50%	99.17%	99.56%
	Recall	99.83%	99.33%	99.66%	

## Data Availability

The data presented in this study are available on request from the corresponding author.
